# Persistent electrical storm following strong acid descaling agent ingestion successfully rescued with venoarterial extracorporeal membrane oxygenation: a case report

**DOI:** 10.1186/s12872-026-05637-8

**Published:** 2026-02-19

**Authors:** Songsong Luo, Wangyan jin, Xiaoyuan Shen, Lujiao Mo, Hongliang Dong, Jiawei Lai

**Affiliations:** Department of Critical Care Medicine, The First People’s Hospital of XiaoshanDistrict, Xiaoshan Affiliated Hospital of Wenzhou Medical University, Hangzhou, 311200 Zhejiang China

**Keywords:** Strong acid ingestion, Electrical storm, Ventricular fibrillation, Cardiogenic shock, Extracorporeal membrane oxygenation, Metabolic acidosis, Hypocalcemia, Corrosive gastritis

## Abstract

**Background:**

Ingestion of industrial strong acids is typically associated with corrosive injury to the gastrointestinal tract and severe metabolic disturbances. While cardiac complications are uncommon, the occurrence of a persistent electrical storm following strong acid ingestion is rarely documented. This case highlights a life-threatening manifestation of non-hydrofluoric acid poisoning, characterized by refractory ventricular arrhythmias and cardiogenic shock, which necessitated advanced extracorporeal life support.

**Case presentation:**

A 62-year-old man accidentally ingested approximately 100 mL of a commercial descaling agent containing hydrochloric and sulfuric acids. He presented with hematemesis, profound metabolic acidosis, and severe ionized hypocalcemia. Approximately two hours after admission, the patient experienced a sudden loss of consciousness due to recurrent ventricular fibrillation. Despite repeated defibrillation, cardiopulmonary resuscitation (CPR), and administration of epinephrine, he experienced a persistent electrical storm and rapidly progressed to refractory cardiogenic shock. Venoarterial extracorporeal membrane oxygenation (VA-ECMO) was initiated as a physiological bridge for circulatory stabilization, alongside continuous renal replacement therapy (CRRT) to proactively manage metabolic triggers. Following the initiation of VA-ECMO, hemodynamic status improved promptly and ventricular arrhythmias ceased. Myocardial function gradually recovered, allowing for successful weaning from extracorporeal support after nine days. Subsequent endoscopy revealed severe corrosive gastritis and pyloric stenosis, managed via laparoscopic jejunostomy. The patient recovered without recurrent arrhythmia and was discharged in improved condition.

**Conclusions:**

In our experience, strong acid ingestion can precipitate malignant ventricular arrhythmias and cardiogenic shock, likely mediated by profound metabolic acidosis and refractory hypocalcemia. This case suggests that caustic acid exposure carries a significant arrhythmogenic risk even in the absence of fluoride toxicity. Our findings indicate that timely escalation to VA-ECMO, combined with aggressive correction of metabolic disturbances, can be lifesaving when conventional therapies fail, providing a critical bridge to recovery for reversible toxin-induced myocardial instability.

**Graphical abstract:**

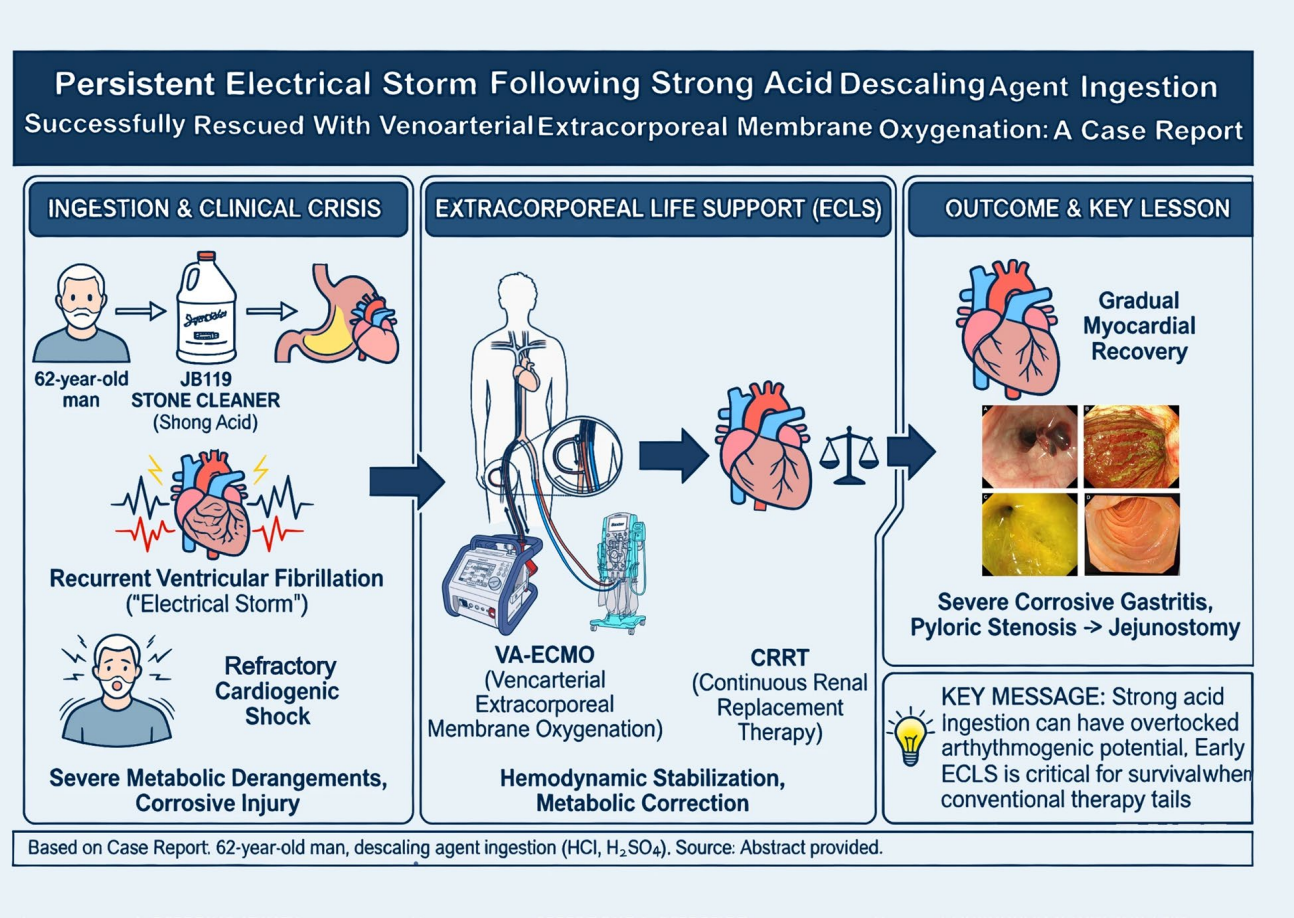

**Supplementary Information:**

The online version contains supplementary material available at 10.1186/s12872-026-05637-8.

## Background

Ingestion of strong industrial acids remains a serious clinical problem worldwide and is most commonly associated with corrosive injury to the upper gastrointestinal tract [1, 2]. The severity of injury depends on the concentration, volume, and duration of exposure, with acids typically causing coagulation necrosis and preferential gastric involvement. In addition to local tissue damage, severe metabolic disturbances, including metabolic acidosis and electrolyte imbalance, may occur and contribute to systemic toxicity [3].

Electrical storm, defined as recurrent episodes of ventricular arrhythmias within a short time frame, is usually associated with structural heart disease, acute myocardial ischemia, or inherited arrhythmia syndromes [4]. Toxicological causes of electrical storm are uncommon, and reports related to caustic acid ingestion are exceedingly rare. The underlying mechanisms are likely multifactorial, involving profound metabolic derangements, electrolyte abnormalities, catecholamine excess, and potentially reversible myocardial dysfunction [5].

Extracorporeal membrane oxygenation has increasingly been used as rescue therapy in cases of refractory cardiogenic shock and life-threatening arrhythmias caused by poisoning or drug intoxication [6]. However, its application in electrical storm following strong acid ingestion has not been well documented. This case therefore aims to highlight a rare arrhythmogenic complication of caustic acid exposure and to emphasize the role of venoarterial extracorporeal membrane oxygenation combined with metabolic support in refractory cases.

## Case presentation

**Fig. 1:**
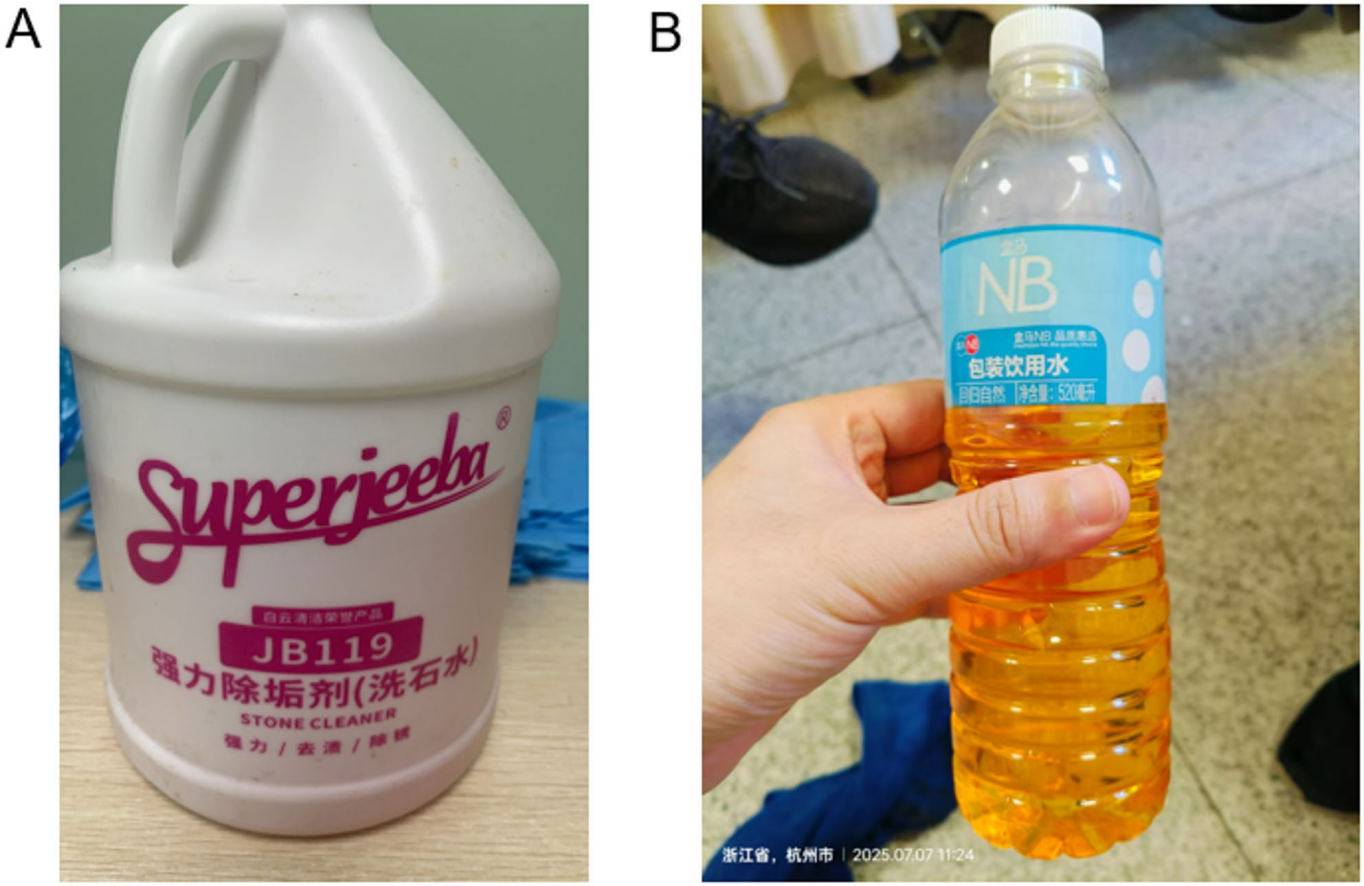
Ingested Cleaning Agent. **A** Packaging of the commercial descaling agent. The agent contains hydrochloric acid, sulfuric acid, and mixed organic acids. **B** Descaling agent placed in a drinking water bottle. The patient accidentally ingested approximately 100 mL of this descaling agent

A 62-year-old man with no known history of cardiovascular disease accidentally ingested approximately 100 mL of a commercial descaling agent containing hydrochloric acid and sulfuric acid (Fig. 1). According to the product information provided by the manufacturer, the descaling agent was confirmed to be free of hydrofluoric acid. He immediately developed severe epigastric pain followed by hematemesis and was transported to the emergency department. He was admitted to our hospital on July 7, 2025. Initial laboratory investigations demonstrated marked metabolic acidosis, hyperchloremia, and severe ionized hypocalcemia. Electrocardiography (ECG) showed atrial fibrillation with rapid ventricular response and QT interval prolongation.

**Fig. 2:**
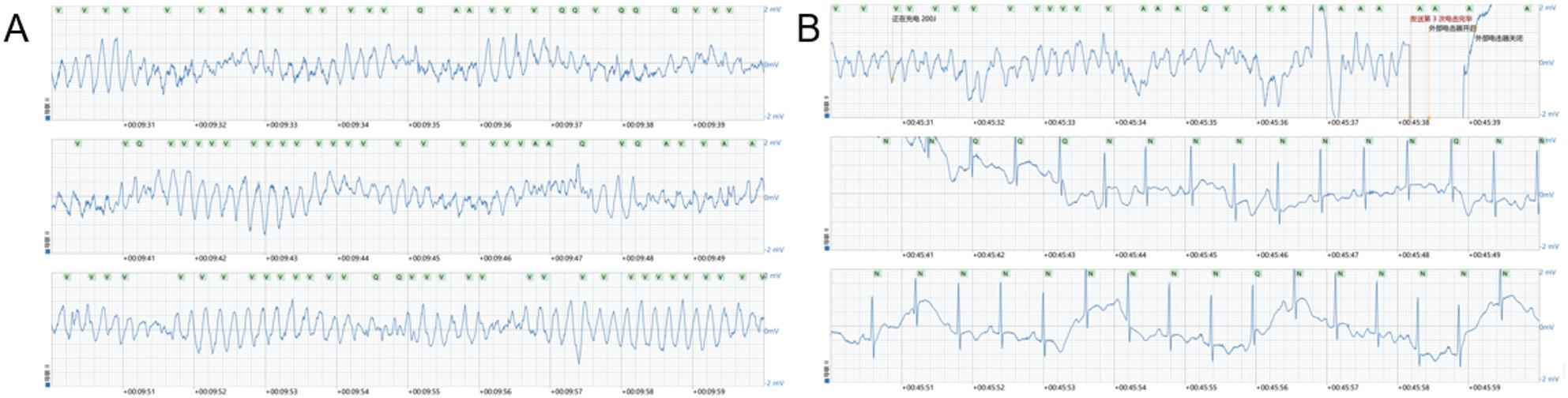
ECG Tracings. **A** Ventricular Fibrillation (VF). The recording displays recurrent episodes of ventricular fibrillation , fulfilling the clinical definition of persistent electrical storm. **B** Defibrillation and Return of Spontaneous Circulation (ROSC). The tracing shows transient return of spontaneous circulation achieved after defibrillation , followed rapidly by the recurrence of ventricular fibrillation

Approximately two hours after admission, the patient experienced sudden loss of consciousness, and cardiac monitoring revealed ventricular fibrillation (Fig. 2A). Although initial defibrillation achieved a transient return of spontaneous circulation (ROSC), recurrent ventricular fibrillation occurred repeatedly within a short period, fulfilling the clinical criteria for a persistent electrical storm [7] (Fig. 2B). This critical resuscitation phase required several rounds of cardiopulmonary resuscitation (CPR), repeated defibrillation, and the administration of epinephrine to maintain circulation. After the final round of resuscitation, the patient eventually achieved a sustained ROSC. However, his condition subsequently deteriorated rapidly into refractory cardiogenic shock with progressive circulatory failure.

However, following ROSC, the patient remained in a state of profound refractory cardiogenic shock with progressive circulatory failure. Prior to the initiation of VA-ECMO, the patient’s hemodynamic status continued to worsen despite maximal conventional support. To maintain systemic perfusion, the norepinephrine infusion was escalated to approximately 3.33 µg/kg/min and epinephrine was administered at 0.35 µg/kg/min. Bedside transthoracic echocardiography demonstrated severely impaired cardiac function, with a left ventricular ejection fraction (LVEF) of approximately 40%, diffuse hypokinesia of the left ventricular wall, and mild left atrial enlargement. Laboratory markers further confirmed profound tissue hypoperfusion; serial arterial blood gas analyses revealed progressive hyperlactatemia accompanied by metabolic acidosis, with lactate levels rising from 7 to 8 mmol/L to 10 mmol/L immediately before VA-ECMO initiation. Venous blood gas analysis showed a markedly reduced central venous oxygen saturation (ScvO_2_ 42.9%). Furthermore, despite continuous supplementation with intravenous calcium gluconate (total dose 5 g), the patient suffered from refractory hypocalcemia, with ionized calcium levels decreasing from 1.0 mmol/L to 0.78 mmol/L just before cannulation.

To prevent further circulatory collapse and provide a bridge to recovery, venoarterial extracorporeal membrane oxygenation (VA-ECMO) was initiated while the patient had a spontaneous heart rhythm. VA-ECMO was established via peripheral cannulation at the bedside, with a 23-Fr venous drainage cannula inserted into the right femoral vein (50 cm) and a 17-Fr arterial return cannula placed in the left femoral artery (23 cm). At initiation, the pump speed was set at 4,000 rpm, achieving a blood flow of approximately 4.2 L/min with a sweep gas flow of 4 L/min. Throughout the support period, lower limb perfusion was assessed daily using bedside ultrasonography and monitoring of the dorsalis pedis artery pulsation; as ultrasound confirmed adequate flow, a distal perfusion catheter was not required.

Following the establishment of VA-ECMO and the subsequent improvement in systemic perfusion, the persistent electrical storm resolved completely, and no further episodes of malignant arrhythmia or requirements for defibrillation occurred during the ECMO run. Continuous renal replacement therapy (CRRT) was started concurrently to facilitate the correction of severe metabolic acidosis and electrolyte disturbances. Serial echocardiography demonstrated progressive recovery of myocardial function.

**Fig. 3:**
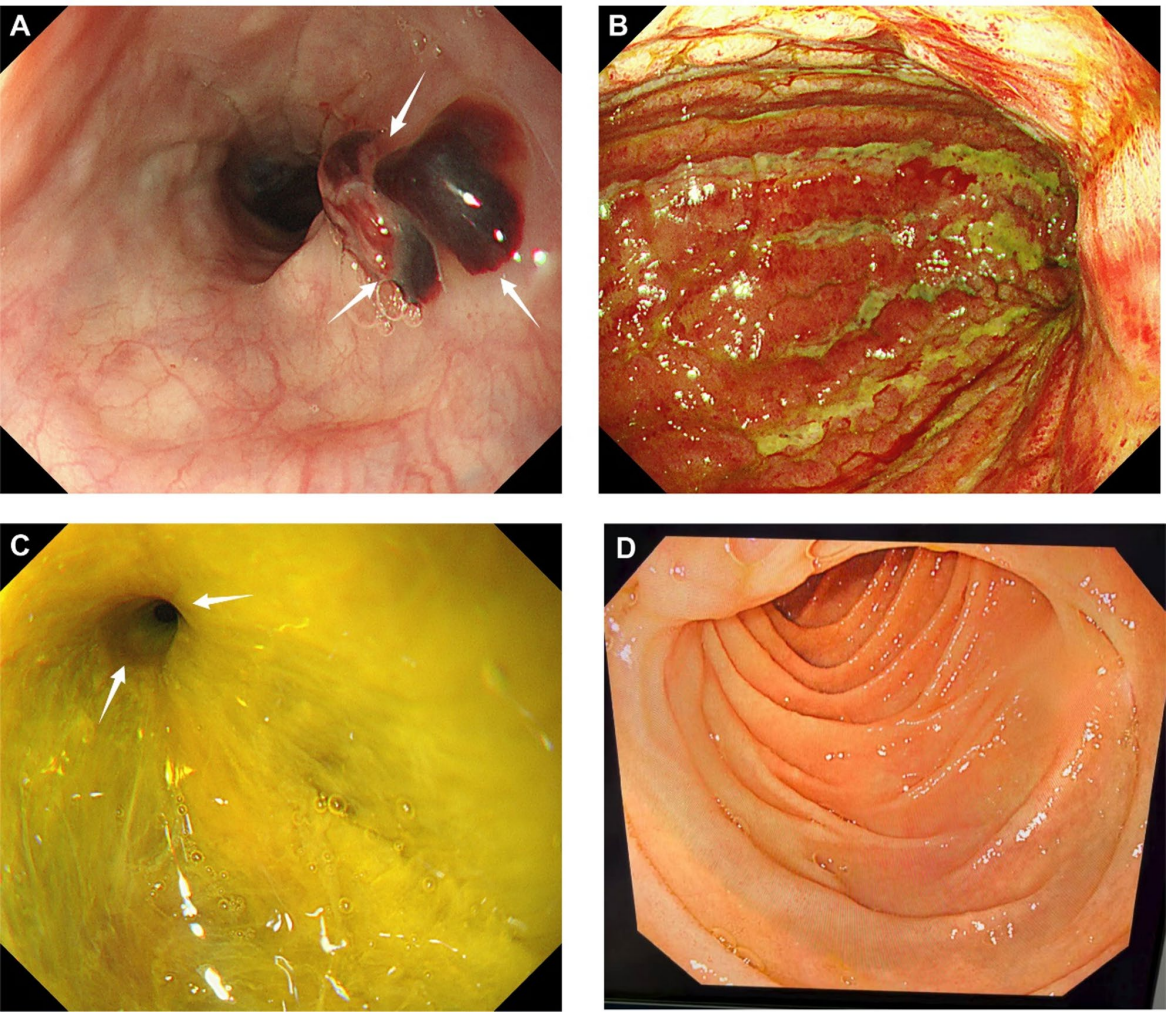
Endoscopic Findings. **A** Esophageal Mucosal Injury. Endoscopy of the esophagus showing a large blood clot (arrows) and erosive injury in the lower esophagus. **B** Corrosive Gastritis. The stomach lining shows severe corrosive gastritis and extensive mucosal necrosis, with the mucosa exhibiting a reddish-green change. **C** Pyloric Stenosis. The gastric lumen contains yellow fluid, and the pylorus (arrows) is markedly stenosed, a typical, delayed evolution consequence of strong acid ingestion. **D** Duodenal Mucosa. The duodenal mucosa appears relatively normal, suggesting the acid injury was mainly confined to the stomach

Given the resolution of the electrical storm and the anticipated short duration of support, the patient was successfully weaned and the VA-ECMO cannulas were removed after nine days. After cardiovascular stabilization, upper gastrointestinal endoscopy revealed severe corrosive gastritis with extensive mucosal necrosis and pyloric stenosis. A laparoscopic jejunostomy was subsequently performed to ensure adequate enteral nutrition (Fig. 3), and the patient was successfully extubated following the surgical procedure. The patient recovered steadily without recurrent arrhythmia and was discharged in improved condition on August 4, 2025, after a total hospital stay of 29 days.

## Discussion

This case broadens the recognized cardiovascular manifestations of strong acid ingestion by demonstrating that malignant ventricular arrhythmias may dominate the early clinical course and critically influence prognosis. Although caustic acid exposure is classically associated with corrosive injury to the upper gastrointestinal tract, severe systemic toxicity may occur when profound metabolic and electrolyte disturbances develop. The occurrence of a persistent electrical storm shortly after ingestion in a patient without pre-existing structural heart disease highlights an underappreciated arrhythmogenic risk of strong acid poisoning.

The pathophysiology underlying electrical storm in this setting is likely multifactorial. Severe ionized hypocalcemia, as observed in this patient, is a well-established contributor to myocardial electrical instability, prolonging repolarization and lowering the threshold for ventricular fibrillation [8]. Metabolic acidosis further exacerbates arrhythmogenesis by altering ion channel function, impairing transmembrane ionic gradients, and reducing myocardial contractility [9]. The combination of hypocalcemia and acidosis may therefore create a highly arrhythmogenic substrate even in structurally normal hearts, predisposing to recurrent ventricular fibrillation [10].

In addition to metabolic factors, transient myocardial dysfunction likely played an important role in the progression to cardiogenic shock. Toxin-induced myocardial depression and stress-related cardiomyopathy have been described in various forms of poisoning and critical illness and are often reversible with appropriate supportive care [11, 12]. Excessive endogenous catecholamine release during acute stress, together with exogenous vasopressor administration during resuscitation, may aggravate myocardial oxygen demand, intracellular calcium overload, and electrical instability, thereby sustaining a vicious cycle of arrhythmia and circulatory collapse. The subsequent recovery of ventricular function in our patient supports the concept of a predominantly reversible myocardial insult rather than irreversible structural damage [13].

Hydrofluoric acid ingestion is a well-recognized cause of malignant ventricular arrhythmias and electrical storm, primarily due to profound hypocalcemia and hypomagnesemia resulting from fluoride ion binding. Numerous reports have documented refractory ventricular fibrillation and sudden cardiac death following hydrofluoric acid exposure, even after ingestion of relatively small amounts. In such cases, aggressive calcium replacement and advanced life support are often required [14–16]. Importantly, the descaling agent ingested by our patient was confirmed to be free of hydrofluoric acid, thereby excluding fluoride toxicity as a contributing mechanism. This distinction is clinically relevant, as it demonstrates that life-threatening electrical storm may also occur following ingestion of strong acids other than hydrofluoric acid. In the absence of fluoride exposure, severe metabolic acidosis and marked hypocalcemia suggest alternative pathways of calcium depletion and myocardial electrical instability. These observations expand current understanding of acid-induced cardiotoxicity and indicate that the arrhythmogenic potential of non–hydrofluoric strong acids may be underestimated.

The management of malignant arrhythmias within the context of toxicological emergencies necessitates a highly individualized approach that is firmly rooted in the specific underlying pathophysiological mechanisms. For instance, poisonings involving glyphosate or organophosphates often present a complex interplay of direct myocardial injury and systemic cholinergic crises, which typically require aggressive extracorporeal blood purification techniques, such as the combination of hemoperfusion and continuous renal replacement therapy, to achieve toxin clearance [17, 18].

In contrast, the ingestion of strong acids like hydrochloric and sulfuric acid presents unique metabolic hurdles that may surpass the capabilities of standard medical interventions. Clinical experience suggests that conventional Advanced Cardiac Life Support (ACLS) protocols including repeated defibrillation and vasopressor administration often prove inadequate when profound metabolic derangements and acute myocardial dysfunction coexist, a phenomenon also observed in severe calcium channel blocker overdoses or mushroom poisonings [19, 20].

In the present case, VA-ECMO served as a vital physiological bridge by providing immediate hemodynamic stabilization. This intervention not only maintained systemic perfusion but also reduced the patient’s requirement for exogenous catecholamines, which can otherwise exacerbate arrhythmogenesis. By establishing this stable circulatory foundation, VA-ECMO created the necessary clinical window to aggressively correct the underlying acid-base imbalances. Consequently, our findings support the early initiation of VA-ECMO as a highly effective strategy for managing refractory electrical storms triggered by corrosive acid ingestion, as it provides the critical time required for both toxin mitigation and complete myocardial recovery [6, 21, 22].

Despite the predominance of cardiovascular manifestations in the acute phase, gastrointestinal injury continued to evolve and required definitive management. The delayed development of pyloric stenosis observed in this patient is consistent with known late complications of acid ingestion and underscores the importance of ongoing surveillance and multidisciplinary care. Close collaboration among intensivists, cardiologists, toxicologists, gastroenterologists, and surgeons is essential to optimize outcomes in such complex cases.

## Conclusion

In our experience, this case highlights that strong acid ingestion, beyond its well-known corrosive effects on the gastrointestinal tract, can trigger a life-threatening, metabolic-driven electrical storm even in patients without pre-existing cardiac disease. The clinical course of our patient suggests that profound metabolic acidosis and refractory hypocalcemia may serve as the primary arrhythmogenic substrate in such poisonings.

While this is a single clinical observation and management should always be individualized, our experience suggests that when conventional antiarrhythmic therapies and advanced cardiac life support fail to stabilize the patient, timely escalation to extracorporeal life support should be considered. The successful recovery of this patient indicates that VA-ECMO, combined with proactive management of metabolic triggers via CRRT, may provide a critical physiological bridge during the reversible phase of toxin-induced myocardial stunning and electrical instability. However, further large-scale studies or registry data are required to validate the broader applicability and optimal timing of VA-ECMO in the context of non-hydrofluoric strong acid poisoning. Ultimately, a multidisciplinary approach remains essential to managing the complex evolution of both systemic cardiovascular crises and delayed gastrointestinal complications.

## Supplementary Information

Supplementary Material 1.

## Data Availability

All data generated or analyzed during this study are included in this published article.

## Abbreviations

CRRT: Continuous renal replacement therapy
ECMO: Extracorporeal membrane oxygenation
VA-ECMO: Venoarterial extracorporeal membrane oxygenation
VF: Ventricular fibrillation

## References

[1] Agarwal A, Srivastava DN, Madhusudhan KS. Corrosive injury of the upper Gastrointestinal tract: the evolving role of a radiologist. Br J Radiol. 2020;93(1114):20200528. 32706982 10.1259/bjr.20200528 PMC7548375

[2] Contini S, Scarpignato C. Caustic injury of the upper Gastrointestinal tract: a comprehensive review. World J Gastroenterol. 2013;19(25):3918–30. 23840136 10.3748/wjg.v19.i25.3918 PMC3703178

[3] Park KS. Evaluation and management of caustic injuries from ingestion of acid or alkaline substances. Clin Endosc. 2014;47(4):301–7. 25133115 10.5946/ce.2014.47.4.301 PMC4130883

[4] Konemann H, Ellermann C, Zeppenfeld K, Eckardt L. Management of ventricular arrhythmias worldwide: comparison of the latest ESC, AHA/ACC/HRS, and CCS/CHRS guidelines. JACC Clin Electrophysiol. 2023;9(5):715–28. 37225314 10.1016/j.jacep.2022.12.008

[5] Challine A, Maggiori L, Katsahian S, Corte H, Goere D, Lazzati A, Cattan P, Chirica M. Outcomes associated with caustic ingestion among adults in a National prospective database in France. JAMA Surg. 2022;157(2):112–9. 34878529 10.1001/jamasurg.2021.6368 PMC8655661

[6] Torre DE, Mangino D, Pirri C. Veno-Arterial Extracorporeal Membrane Oxygenation in Cardiotoxic Drug-Induced Cardiogenic Shock: A Systematic Narrative Review. Life. 2025;15(6):925. 10.3390/life15060925. 10.3390/life15060925 PMC12194164 40566578

[7] Elsokkari I, Sapp JL. Electrical storm: prognosis and management. Prog Cardiovasc Dis. 2021;66:70–9. 34332662 10.1016/j.pcad.2021.06.007

[8] Vandewiele F, Pironet A, Jacobs G, Kecskes M, Wegener J, Kerselaers S, Hendrikx L, Verelst J, Philippaert K, Oosterlinck W, et al. TRPM4 Inhibition by meclofenamate suppresses Ca2+-dependent triggered arrhythmias. Eur Heart J. 2022;43(40):4195–207. 35822895 10.1093/eurheartj/ehac354

[9] Jentzer JC, Noseworthy PA, Kashou AH, May AM, Chrispin J, Kabra R, Arps K, Blumer V, Tisdale JE, Solomon MA, et al. Multidisciplinary critical care management of electrical storm: JACC State-of-the-Art review. J Am Coll Cardiol. 2023;81(22):2189–206. 37257955 10.1016/j.jacc.2023.03.424 PMC10683004

[10] Wigginton JG, Agarwal S, Bartos JA, Coute RA, Drennan IR, Haamid A, Kudenchuk PJ, Link MS, Panchal AR, Pelter MM, et al. Part 9: adult advanced life support: 2025 American heart association guidelines for cardiopulmonary resuscitation and emergency cardiovascular care. Circulation. 2025;152(16suppl2):S538–77. 41122884 10.1161/CIR.0000000000001376

[11] Lavonas EJ, Akpunonu PD, Arens AM, Babu KM, Cao D, Hoffman RS, Hoyte CO, Mazer-Amirshahi ME, Stolbach A, St-Onge M, et al. 2023 American heart association focused update on the management of patients with cardiac arrest or Life-Threatening toxicity due to poisoning: an update to the American heart association guidelines for cardiopulmonary resuscitation and emergency cardiovascular care. Circulation. 2023;148(16):e149–84. 37721023 10.1161/CIR.0000000000001161

[12] Pozzi M, Buzzi R, Hayek A, Portran P, Schweizer R, Fellahi JL, Armoiry X, Flagiello M, Grinberg D, Obadia JF. Veno-arterial extracorporeal membrane oxygenation for drug intoxications: A single center, 14-year experience. J Card Surg. 2022;37(6):1512–9. 35353389 10.1111/jocs.16456

[13] Compagnon B, Tardif E, Pey V, Marcheix B, Labaste F, Conil JM, Minville V, Vardon-Bounes F. Use of extracorporeal membrane oxygenation in severe cardiotoxic poisoning: analysis of a cohort over 10 years. Sci Prog. 2025;108(4):368504251358951. 41032584 10.1177/00368504251358951 PMC12489209

[14] Kroes JA, de Haan JMH, de Haan-Lauteslager MI, van Roon EN, Derksen SJ, Manusama ER, Zijlstra GJ, Gisbertz SS, Vrijsen BEL, Bethlehem C. Delayed cardiac arrest after hydrofluoric acid ingestion. Clin Toxicol (Phila). 2024;62(3):205–7. 38501538 10.1080/15563650.2024.2328348

[15] Ramesh S, Nathan B, Vt A, Gaayathri M. Hydrofluoric acid fatality from dermal exposure. J Emerg Med. 2025;79:453–6. 41205306 10.1016/j.jemermed.2025.09.033

[16] FarkasAN, Wolf MS, Landzberg E, Lynch MJ, Woods KS. Treatment of Ventricular Fibrillation Due to Ammonium Bifluoride Poisoning With Hemodialysis. Pediatrics. 2018;142(3):e20180136. 10.1542/peds.2018-0136. 10.1542/peds.2018-0136 30111553

[17] Guo Y. N <>Li 2024 Network toxicology and molecular Docking to investigative the non-acetylcholinesterase mechanisms and targets of cardiotoxicity injury induced by organophosphorus pesticides. Medicine 103 41 e39963. 39465796 10.1097/MD.0000000000039963 PMC11479526

[18] Ge X, Yang Z, Cai Q. The capillary-leakage syndrome caused by glyphosate poisoning: a case report. Annals Med Surg (2012) 2023, 85(4):1180–3. 10.1097/MS9.0000000000000393 PMC10129225 37113950

[19] Ou L, Lin J, Zhang H, Xu X, Lai S. Experience and reflection on the treatment of calcium channel blockers poisoning: two case reports of elderly patients. Clin Case Rep *2025*, 13(11):e71374. 10.1002/ccr3.71374 PMC12634312 41280266

[20] Zhang Y, Fu Y, Zhu H, Luo Z, Zhong L, Lin J, Wu X, Cao X, Deng W, Liu W, Yang Z. A Retrospective Analysis of 112 Mushroom Poisoning Patients - Guangzhou City, Guangdong Province, China, 2016–2023. *China CDC weekly* 2025, 7(19):665-671.21. 10.46234/ccdcw2025.110 PMC12075496 40376181

[21] Ward C, Meeks D, Trimlett R, Alcada J. Taxine alkaloid poisoning successfully supported with venoarterial extracorporeal membrane oxygenation: a case report. Eur Heart J Case Rep. 2022;6(2):ytac039. 35187392 10.1093/ehjcr/ytac039 PMC8851931

[22] Kumar A, Prakash J, Berwal K, Arya G, Narwal V, Arora E, Kumar A, Yadav N, Chaudhry D, Dhankar A, et al. Real-world experience of veno-arterial extracorporeal membrane oxygenation in severe aluminium phosphide poisoning. Clin Toxicol (Phila). 2025;63(11):878–86. 40874872 10.1080/15563650.2025.2544948

